# Draft Genome Sequence of the *Xanthomonas cassavae* Type Strain CFBP 4642

**DOI:** 10.1128/genomeA.00679-13

**Published:** 2013-08-29

**Authors:** Stéphanie Bolot, Alejandra Munoz Bodnar, Sébastien Cunnac, Erika Ortiz, Boris Szurek, Laurent D. Noël, Matthieu Arlat, Marie-Agnès Jacques, Lionel Gagnevin, Perrine Portier, Marion Fischer-Le Saux, Sébastien Carrere, Ralf Koebnik

**Affiliations:** INRA, Laboratoire des Interactions Plantes Micro-organismes (LIPM), UMR 441, Castanet-Tolosan, Francea; CNRS, LIPM, UMR 2594, Castanet-Tolosan, Franceb; UMR 186 IRD-Cirad-Université Montpellier 2 *“*Résistance des Plantes aux Bioaggresseurs,*”* Montpellier, Francec; Université de Toulouse, Université Paul Sabatier, Toulouse, Franced; INRA, UMR1345 Institut de Recherche en Horticulture et Semences, Angers, Francee; Agrocampus Ouest, UMR1345 Institut de Recherche en Horticulture et Semences, Angers, Francef; Université d’Angers, UMR1345 Institut de Recherche en Horticulture et Semences, SFR4207 QUASAV, PRES L’UNAM, Angers, Franceg; UMR *“*Peuplements Végétaux et Bioagresseurs en Milieu Tropical*”* (PVBMT), CIRAD, Saint-Pierre, La Réunion, Franceh; INRA, CIRM-CFBP Collection Française de Bactéries associées aux Plantes, Angers, Francei

## Abstract

We report the draft genome sequence of the *Xanthomonas cassavae* type strain CFBP 4642, the causal agent of bacterial necrosis on cassava plants. These data will allow the comparison of this nonvascular pathogen with the vascular pathogen *Xanthomonas axonopodis* pv. manihotis, both infecting the same host, which will facilitate the development of diagnostic tools.

## GENOME ANNOUNCEMENT

Cassava (*Manihot esculenta* Crantz) is the third most important source of calories in the tropics, after rice and maize, and millions of people in Africa, Asia, and Latin America depend on cassava. South America, probably the Amazon region, is considered the center of origin for the cassava species. It was only in the 16th century that Portuguese navigators introduced cassava to the west coast of Africa, from where it later disseminated to East Africa. Most of the spread within the African continent, however, took place only during the 20th century due to colonial powers encouraging its cultivation (1). Nowadays, cassava is grown in all Sub-Saharan countries, and Africa produces more cassava than the rest of the world combined (1). Cassava plants are frugal with respect to environmental conditions (drought, poor soil) and hold great promise as a future staple crop in Africa, since this species might not only tolerate but even profit from climate change (2).

Cassava plants can suffer from two bacterial diseases, bacterial blight and bacterial necrosis, caused by two species of *Xanthomonas*. The causal agent of bacterial blight, *X. axonopodis* pv. manihotis, is a vascular pathogen which has been well studied over the last years (3). Recently, draft genome sequences of 65 strains have been elucidated (4). Much less is known about the nonvascular pathogen *X. cassavae*, which causes bacterial necrosis in Africa (5). The comparison of a vascular and a nonvascular pathogen, both infecting cassava, might give important clues about determinants of tissue specificity during colonization of the host plant. This prompted us to sequence the *X. cassavae* type strain CFBP 4642 (NCPPB 101, ICMP 204, LMG 673), which was isolated in Malawi in 1951.

Type strain CFBP 4642 was sequenced using the Illumina Hi-Seq2000 platform (GATC Biotech, Germany). The shotgun sequencing yielded 95,437,238 read pairs (64,851,255 100-bp paired-end reads with an insert size of 250 bp and 30,585,983 50-bp mate-pair reads with an insert size of 3 kb). A combination of Velvet (6), SOAPdenovo, and SOAPGapCloser (7) yielded 83 contigs larger than 500 bp (*N*_50_, 158,383 bp) with the largest contig of 425 kb for a total assembly size of 5,263,056 bp.

Multilocus sequence analysis of four housekeeping genes described earlier for xanthomonads (8) revealed that the four internal fragments are 99.94% identical (3,371/3,373) to those of the *X. cassavae* type strain ICMP 204 at PAMDB (9). The genome encodes a canonical type III protein secretion system (10) and several type III effectors, such as transcriptional activator-like (TAL) effectors (5). Interestingly, a CRISPR/*cas* defense system is present, which might be exploited to develop a powerful tool for Pan-African epidemiological surveillance (11).

### Nucleotide sequence accession number. 

This whole-genome shotgun project has been deposited in GenBank under the accession no. ATMC00000000.
